# Carbohydrate based biomarkers enable hybrid near infrared fluorescence and ^64^Cu based radio-guidance for improved surgical precision

**DOI:** 10.7150/ntno.60295

**Published:** 2021-05-17

**Authors:** Wenbo Wang, Anders E. Hansen, Hongmei Sun, Frederikke P. Fliedner, Andreas Kjaer, Andreas I. Jensen, Thomas L. Andresen, Jonas R. Henriksen

**Affiliations:** 1 Technical University of Denmark, Department of Health Technology, Building 423, 2800 Lyngby, Denmark.; 2 Department of Clinical Physiology, Nuclear Medicine & PET and Cluster for Molecular Imaging, Department of Biomedical Sciences, Rigshospitalet and University of Copenhagen, Blegdamsvej 9, DK-2100 Copenhagen, Denmark.; 3 School of Bioengineering and Food, Key Laboratory of Fermentation Engineering, (Ministry of Education), Key Laboratory of Industrial Microbiology in Hubei, National '111' Center for Cellular Regulation and Molecular Pharmaceutic, Hubei province Cooperative Innovation Center for Industrial Fermentation, Hubei University of Technology, Wuhan 430068, China; 4 Technical University of Denmark, The Hevesy Laboratory, Department of Health Technology, 4000 Roskilde, Denmark; 5 Center for Nanomedicine and Theranostics, Technical University of Denmark, 2800 Lyngby, Denmark.

**Keywords:** Surgical marker, Near infrared fluorescence, PET/CT, Naphthalocyanine, Cu-64

## Abstract

Increasing numbers of lung tumors are identified at early disease stages by diagnostic imaging in screening programs, but difficulties in locating these during surgical intervention has prevented an improved treatment outcome. Surgical biomarkers that are visible on diagnostic images, and that provide the surgeon with real-time image guidance during the intervention are thus highly warranted to bridge diagnostic precision into enhanced therapeutic outcome. In this paper, a liquid soft tissue marker for near infrared fluorescence and radio-guidance is presented. The biocompatible marker is based on the carbohydrate ester, sucrose acetate isobutyrate, ethanol, and a multifunctional naphthalocyanine dye, which enable near infrared fluorescence image-guided resection at short, medium and long tissue depths. Naphthalocyanine dyes have high quantum yields and may further act as chelators of radionuclides. Upon injection of the liquid marker, a gel-like depot is formed *in situ* at the site of injection, wherein the fluorescent dye and radionuclide is retained. The radiolabeled markers were optimized for minimal fluorescence quenching and high retention of the positron emission tomography radionuclide ^64^Cu. The performance of the radiolabeled marker was tested *in vivo* in mice, where it displayed high photostability over a period of 4 weeks, and high retention of ^64^Cu for 48 hours. The retention and biodistribution of ^64^Cu was quantified via PET/CT, and the fluorescence emission by an *in vivo* imaging system. The presented data demonstrate proof-of-concept for naphthalocyanine markers as multimodal imaging agents that can bridge the precision of diagnostic imaging into surgical interventions.

## Introduction

**Figure 1:**
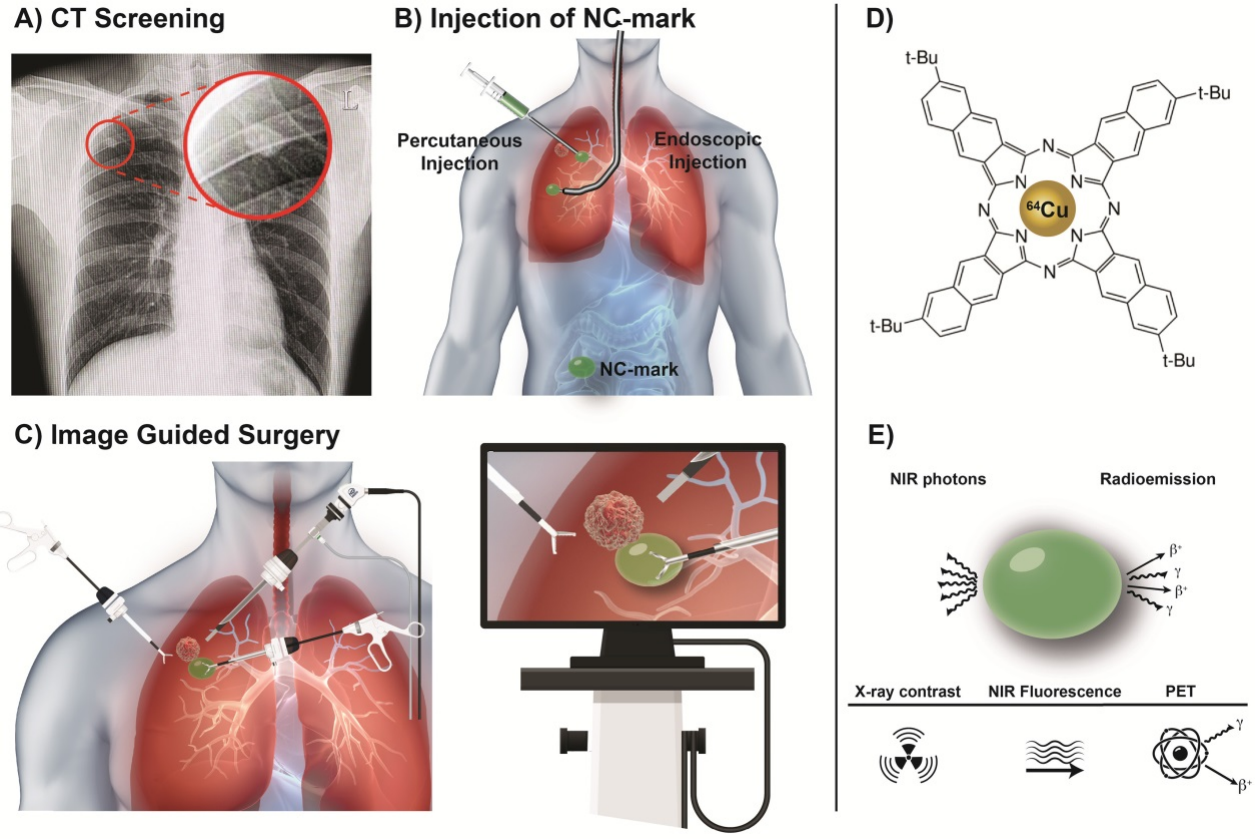
**Video-Assisted Thoracic Surgery** (**VATS), guided by a soft tissue marker.** (A) A small pulmonary nodule is identified via CT screening (highlighted by red circle). (B) Prior to surgery a multimodal liquid marker (NC-mark), containing a naphthalocyanine dye, is injected next to the lesion, using endoscopy or percutaneous administration. (C) The patient then undergoes a VATS procedure where the cancer lesion is identified via the position of the multimodal marker that is located using a NIR camera or a small gamma probe. (D) NC-mark contains a multifunctional naphthalocyanine dye that also act as a chelator of ^64^Cu. (E) Emission of NIR photons by fluorescence or positrons and gamma emission from ^64^Cu enables real-time image guidance for the location of NC-mark. X-ray contrast provided by the iodinated carbohydrate ester xSAIB provides visibility of NC-mark in CT and fluoroscopy imaging.

Lung cancer is a leading cause of cancer-related deaths and patients diagnosed at late disease stages often face a very poor prognosis 1. The emergence of screening programs and improvement in resolution and sensitivity of diagnostic imaging technologies has increased the number of cases where small-sized solitary pulmonary nodules (SPNs) are identified (Figure 1A). Such SPNs should ideally be surgically resected at the time of diagnosis to prevent progression of disease. However, the small size or potential distance from the lung surface makes the majority of SPNs non-palpable and therefore often impossible for the surgeon to locate and resect. Surgical intervention may in such cases be postponed until the nodule has grown to a palpable size or other measures are taken. These delays are unsatisfactory for patients and increase the risk of disease progression, which worsens prognosis and increases treatment associated costs. Markers that can be accurately positioned in or adjacent to the tumor and provide an easily identifiable object, or signal, during surgical interventions are therefore highly desired (Figure 1B). Such markers would allow the surgeon to resect otherwise undetectable SPNs and thereby increase surgical sensitivity and specificity. Video-assisted thoracic surgery (VATS, Figure 1C) has become the primary method for thoracic pulmonary surgery, as it is efficacious, less invasive, lowers the risk of adverse events, increases the chance of safe resection, and reduces patient discomfort 2. The optimal marker should therefore be able to bridge the gap between the accuracy of modern diagnostic imaging and treatment by surgery. Such markers are therefore intensively explored for preoperative and intraoperative target localization and navigation. Examples of currently available markers for surgical guidance include hook wires, iodinated oils (Lipiodol) and ^99m^Tc-labeled nanoparticles that are visible in ultrasonography, CT or SPECT imaging, respectively 3-5. Marker-associated complications are however common, e.g. hook wires are considered unpleasant and painful for patients and may potentially migrate prior to, or during surgery 6. Lipiodol mainly provides preoperative information, causes radiation exposure, and has been reported to cause systemic embolization 6. ^99m^Tc-labeled nanoparticles can be identified during surgery via hand-held gamma detectors and preoperatively by SPECT imaging, but the marker may disperse in the tissue upon injection and accuracy is lost over time, which may misguide the surgeon 5. Common flaws for these markers are their inability to be identified across a range of medical imaging modalities, lack of positional stability and poor patient compliance.

Consequently, it is desirable to develop surgical markers that are (i) minimally invasive to patients, (ii) visible on diagnostic imaging, (iii) easy to locate during surgery at any tissue depth, and (iv) that are positionally stable after placement. For short range surgical guidance, near-infrared (NIR) fluorescence imaging is attracting increasing interest, due to its high spatial and temporal resolution 7. Currently, two FDA-approved dyes, methylene blue (MB) and indocyanine green (ICG), are clinically used for fluorescence-guided interventions, including sentinel lymph node mapping and image-guided surgery 8-12. Both dyes were developed for systemic administration and consequently have poor retention after direct injection in tissue. The penetration depth of fluorescence in tissues is limited to the cm-range 13 due to scattering and absorption of the incoming and emitted NIR light 14. Surgical guidance beyond the cm-range can be realized by combination of NIR imaging with e.g. radio-guidance, which is highly relevant for surgical guidance in e.g. robot-assisted surgery 15, 16.

**Figure 2:**
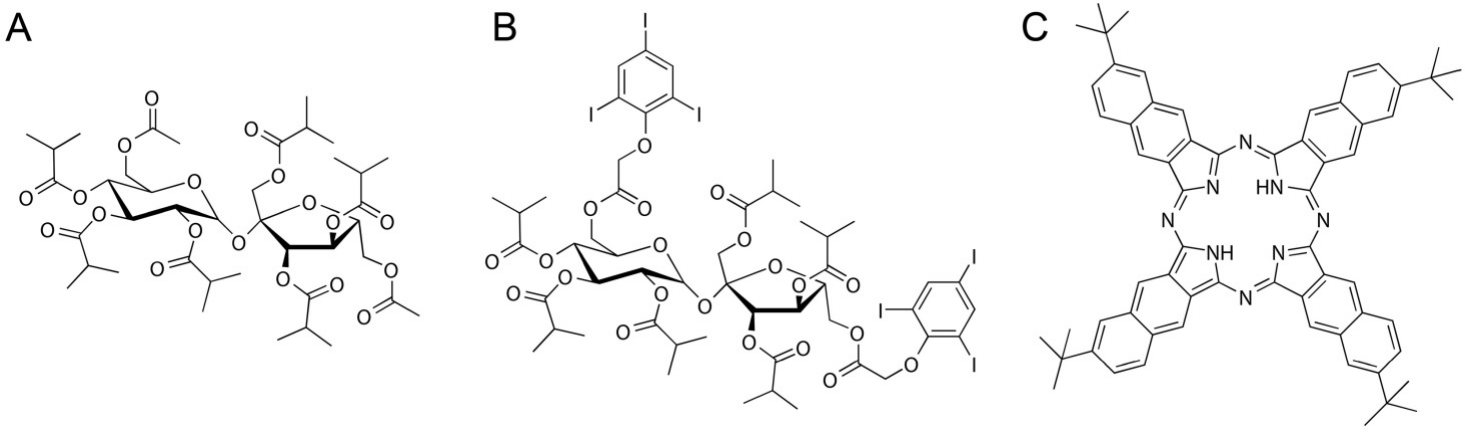
** Chemical structures of NC-mark constituents.** SAIB (A), xSAIB (B) and 2,11,20,29-tetra-tert-butyl-2,3-naphthalocyanine (NC) (C).

The accuracy of modern image-guided injection and aspiration technologies allows for marker installation directly in the tissue of interest with low risks of injection-associated side-effects. Lower rates of pneumothorax have as an example been reported for bronchoscopy assisted injections 17. This has motivated the development of liquid *in situ* forming markers based on sucrose acetate isobutyrate (SAIB, Figure 2A) for soft tissue injection as an alternative to the intravenously administered probes 18. In one example, a SAIB-based marker for image-guided radiotherapy was developed, and demonstrated good visibility, size- and positional stability during a 7 weeks course of fractionated radiation therapy in lung cancer patients 19. Additionally, a ^125^I-radiolabeled SAIB derivative has been synthesized and embedded in a SAIB-based marker, providing both radiographic contrast and possible gamma camera detection 20.

In the current study, a liquid, injectable, soft tissue marker, intended for fluorescence- and or radio-guided surgery, is presented. The marker is designed to form a gel-like depot at the injection site and is based on SAIB and ethanol as co-solvent. The formulation further contains a naphthalocyanine dye (2,11,20,29-tetra-tert-butyl-2,3-naphthalocyanine, NC, Figure 2C, Figure 1D) that provides the double functionality of the marker. NC is part of a family of intensely colored symmetric aromatic macrocyclic structures, such as phthalocyanine and porphyrins, that all have high photostability and quantum yields 21. Depending on chemical substituents, the absorption and fluorescence emission properties of these dyes can be tuned in the range from 300 to 900 nm. Phthalocyanines and naphthalocyanines are therefore used in photodynamic therapy, imaging, theranostics 22-24 and for fluorescence based surgical guidance. These macrocycles further form complexes with a range of metals including Cu, Ni, Co, Zn etc. 25, which in the current work is utilized for radiolabeling of the marker, providing options for deep tissue localization. Naphthalocyanine has previously been successfully radiolabeled with ^64^Cu as part of a multimodal micelle construct 26, 27, which enable PET imaging and localization using high energy gamma detectors. Surgical guidance using PET radionuclides has further been validated in clinical studies 28. All constituents of these markers are acceptable for clinical use, as SAIB has been approved by the FDA as generally-recognized-as-safe (GRAS), and phthalocyanine/naphthalocyanine dyes are currently being tested in clinical trials for photodynamic therapy 29. Due to the low viscosity of the SAIB-ethanol mixtures 30 these liquid markers can be injected using thin needle technologies (Figure 1C). The liquid marker is further compatible with state-of-the-art interventional radiology and image-guided technologies e.g. electromagnetic navigation bronchoscopes 31. This enables high precision placement at the site of interest in basically all tissue accessible for fine needle injection or aspiration. Upon injection in soft tissue, ethanol diffuses out of the marker, resulting in *in situ* formation of a highly viscous, gel-like depot 18, 30. This increase in marker viscosity minimizes the risk of marker migration, and secures optimal retention of marker constituents, which reduces the leaching of fluorophore and radionuclides from the injection site. Moreover, the enhanced viscosity of the material provides strong ultrasound reflections, rendering the material highly visible in ultrasonography 32. The marker further contains the radiographic contrast agent xSAIB, an iodine-rich sugar ester (Figure 2B). The clinician may therefore employ ultrasonography, 2D x-ray fluorescence or CT guidance during injection for optimal positioning of the marker.

In the current study, the fluorescence intensity of the new soft tissue marker (NC-mark) was optimized for minimal self-quench, and the marker was radiolabeled with the PET radionuclide ^64^Cu (T_½_ = 12.7 h) for positron emission tomography (PET) or detection via positron emission derived annihilation photons (511 keV) using high energy gamma detectors 28. The fluorescence properties and marker retention of ^64^Cu were quantified in bench and *in vivo* testing. The dual functional NC dye employed in this study displayed strong image features for guided surgical resection at short, medium and long distances by inclusion of a single multifunctional compound.

## Experimental section

### Material

6,6'-di-triidobenzene-isobuturic-sucrose (xSAIB) was custom synthesized 33. The freeze dried, premixed stealth liposome mixture of hydrogenated soy phosphatidylcholine (HSPC), cholesterol (CHOL) and 1,2-distearoyl-sn-glycero-3-phospho-ethanolamine-N-[methoxy(polyethylene glycol)-2000] (DSPE- PEG2000) (565:382:53, molar ratio) was purchased from Lipoid. Sucrose acetate isobutyrate (SAIB), cupric chloride dihydrate (CuCl_2_ · 2H_2_O), 2,11,20,29-Tetra-tert-butyl-2,3-naphthalocyanine (NC) and all other chemicals were purchased from Sigma Aldrich. All the chemicals and reagents were of analytical grade and used without further purification.

The UV-vis spectra of marker formulations were recorded by a multimode microplate reader (Tecan). The fluorescence emission of markers with NC was measured by a fluorescence spectrometer (OLIS SLM8000 or OLIS DM 45) in quartz cuvettes (Helma). Surface fluorescence imaging was recorded by using an Odyssey FC imaging system (Licor).

Radioactivity was measured by a Veenstra Instruments dose calibrator VDC-505 (Comecer) or by liquid scintillation counting on a 300 SL spectrometer (HIDEX). The scintillation vials and the Ultima Gold scintillation cocktail were purchased from PerkinElmer. All radio-thin layer chromatography (radio-TLC) was performed on silica gel 60 F254 plates (Merck), using CHCl_3_:MeOH:AcOH (98:1:1) as eluent. A MiniGita Star with a Beta Detector GMC probe (Perkin-Elmer) was used for analysis of radio-TLCs with radioactive peaks integrated using associated computer software.

Isotonic buffer, ISO-TRIS, containing 2-amino-2-(hydroxymethyl)-1,3-propanediol (TRIS, 10 mM) and sodium chloride (150 mM), was prepared and adjusted to a final pH of 7.8, using hydrochloric acid (0.1 M). Phosphate-buffered saline was prepared by mixing sodium phosphate monobasic dihydrate and sodium phosphate dibasic dihydrate to obtain pH 7.4 (10 mM phosphate, 150 mM NaCl, pH 7.4).

The mini-extruder was purchased from Avanti Polar Lipids. The hydrodynamic size of liposomes was measured in ISO-TRIS by dynamic light scattering (DLS) on a Zetasizer (Malvern). Phosphor concentration of liposomes was measured by ICP-MS (Thermo Scientific, iCAP Q).

### Preparation of the NC-mark formulations

A marker matrix comprising SAIB:xSAIB:ethanol 70:10:20 (w/w) was prepared by simple mixing. SAIB (7.0 g) was heated to 70 ℃ and poured into a glass vial. xSAIB (1.0 g) and ethanol (2.0 g) were mixed with the SAIB, and the combined mixture was sonicated for 30 minutes to obtain a transparent and homogeneous marker solution.

A NC-mark formulation with 0.1 % (w/w) NC was prepared. PC (1.0 mg) was weighted into a glass vial. To this vial was added marker solution (SAIB:xSAIB:ethanol 70:10:20, 1.0 g) and the resulting mixture was sonicated at 55 ℃ for 6 hours, followed by magnetic stirring at 55 ℃ for 16 hours. Markers with different NC concentrations (0.05, 0.01, 0.006, 0.003, 0.001, 0.0006, 0.0003, 0.0001 or 0.00001 %, w/w) were prepared by diluting the 0.1% NC-mark formulation with marker matrix. The resulting markers were homogenized by 5 minutes of magnetic stirring at room temperature.

### UV-vis spectra of markers

Markers with different NC concentrations (0.01, 0.006, 0.003, 0.001, 0.0006, 0.0003, 0.0001 or 0.00001 % w/w) were prepared as described above. Each marker (0.2 mL) was pipetted into a 96-well plate, and the UV-vis spectrum (400-1000 nm) was recorded by a multimode microplate reader with a bandwidth of 3.5 nm.

### Fluorescence emission

The fluorescence spectra of markers with different NC concentrations (0.1, 0.05, 0.01, 0.006, 0.003, 0.001, 0.0006, 0.0003, 0.0001 or 0.00001 %) were measured using a fluorescence spectrometer (OLIS SLM8000). Briefly, each marker solution (1.2 mL) was transferred to a quartz cuvette, and the fluorescence emission spectra were recorded in a wavelength range of 780-830 nm at an excitation wavelength of 768 nm, scan time of 45 seconds and a slit width of 8 nm. The full fluorescence spectrum of the marker with NC (0.001 %, w/w) was measured using a fluorescence spectrometer (OLIS DM 45). Briefly, each marker solution (1.2 mL) was transferred to a quartz cuvette and the fluorescence emission spectra was recorded in a wavelength range of 780-830 nm at an excitation wavelength of 700 nm with an integration time of 0.2 seconds and a slit width of 26 nm.

### Surface fluorescence imaging and the influence of ethanol on fluorescence

NC-mark formulations containing different NC concentrations (70 µL, 0.01, 0.006, 0.003, 0.001, 0.0006, 0.0003, 0.0001 or 0.00001 %) were spotted on a 10-well cover glass. The surface fluorescence of the markers was measured by an *in vitro* NIR system (Licor Odyssey^®^ Fc Imaging System, excitation wavelength at 785 nm, emission wavelength at 800 nm, and spatial resolution of 125 µm). Following, the 10-well cover glass with markers was stored in a vacuum oven at 55 ℃ (P < 10 mBar) overnight in order to remove ethanol from the markers. After the markers had cooled to room temperature, the surface fluorescence was remeasured following the same procedure as described previously.

### Effect of copper on the fluorescence of markers

A solution of CuCl_2_ · 2H_2_O in ethanol (0.005 mg/mL) was prepared and transferred to glass vials (0, 43.6, 87.1, 130.7, 217.8 or 435.6 µL). The ethanol in each vial was evaporated by heating at 55 ℃ under a flow of nitrogen. NC-mark formulations (1.2 mL, 0.001 % NC) was added to each vial containing different amounts of CuCl_2,_ resulting in molar ratios of Cu^2+^ / NC of 0, 1:10, 1:5, 3:10, 1:2 and 1:1, respectively. The resulting mixtures were magnetically stirred at 55 ℃ for 2 hours, allowing copper to form a chelate with the NC. The UV-vis absorbance and the fluorescence emission spectra of each mixture was measured as previously described.

### Preparation of ^64^Cu

^64^Cu was produced on a PETtrace cyclotron (GE Healthcare) equipped with a beamline by proton irradiation of an electroplated ^64^Ni target, then purified by anion exchange chromatography in aqueous hydrogen chloride (HCl) media. The ^64^Cu was ultimately obtained in aqueous HCl (1.0 M), and isolated by evaporation of aqueous HCl by argon flow, as described before 34. The dry ^64^CuCl_2_ was used for radiolabeling markers.

### Radiolabeling of markers

NC-mark formulation (750 µL, SAIB:xSAIB:EtOH:NC, 70:10:20:0, 70:10:20:0.001 or 70:10:20:0.01) was added to dry ^64^CuCl_2_ (150 MBq). The resulting mixtures were magnetically stirred at 55 ℃ for 2 hours. A small amount of each radiolabeled marker solution was weighed into a glass vial and dissolved in acetonitrile to a concentration of about 10 mg marker/mL. The resulting solution was analyzed by radio-TLC. The formation of ^64^Cu-NC was confirmed by comparing the obtained TLC retention factor (R*f* = around 0.8) with that of a non-radioactive chemically identical reference compound (Supporting Information (SI), S1). The R*f* of ^64^Cu in markers not containing NC remained at the origin (R*f* = 0).

The non-radioactive reference complex was produced by adding a chloroform solution of NC (1 mL, 1.0 mg/mL) to CuCl_2_·2H_2_O (0.02 mg) for a Cu:NC molar ratio of 10:1. The resulting mixture was magnetically stirred at 55 ℃ for 2 hours. The Cu-NC complex and NC were analyzed by MALDI-TOF MS (Bruker Reflex, Bruker Daltonics, Billerica, MA, USA): Calc. M: 939.2 Da., Obs. M: 939.2 Da. (PC dye). Calc. M: 1000.7 Da., Obs. M: 1000.0 Da (Cu-NC complex). 1 µL of the mixture was spotted on silica gel 60 F254 plates (Merck) and a solution of CHCl_3_:MeOH:AcOH 98:1:1 was used as eluent. The R*f* of the resulting Cu-NC complex was about 0.8.

### *In vitro* release of ^64^Cu from NC-markers

Radiolabeled NC-markers (100 µL, 0, 0.01 or 0.001 %, w/w) with an initial radioactivity concentration of 300 MBq/mL were injected into release medium (4.0 mL), which contained ISO-TRIS, EDTA (1.0 mM) and stealth liposomes (lipid concentration: 5.0 mM). Liposomes were produced by hydrating commercial stealth lipid mixture with ISO-TRIS (37.5 mg/mL) at 65 ℃ by sonication for 1 hour followed by sizing with a mini-extruder with a cut-off size of 200 nm. The size of the liposomes was 143 ± 2 nm with a PDI of 0.19 ± 0.01. The phosphor concentration of the liposomes was measured using ICP-MS with an internal standard (gallium, 10 ppb). The radioactivity of each marker injected into the buffer was measured on a dose calibrator. The *transfer efficiency*, defined as the ratio of the measured radioactivity to the theoretical radioactivity of the marker that was injected in the buffer, was calculated for each formulation.

Aliquots (15-1000 µL) of release medium were removed as a function of time (1 hour, 3 hours, 6 hours, 1 day, 2 days, 4 days and 6 days), and replaced with an equal amount of fresh release medium. After 6 days, all the release medium was removed, and the remaining marker was dissolved in ethanol (1.0 mL). An aliquot of the resulting solution (250 µL) was removed for quantification. All removed aliquots were analyzed by liquid scintillation. A calibration curve (20-800 Bq) was prepared for ^64^Cu and was linear in the required concentration range (*r*^2^ > 0.999).

### *In vivo* experiments

NC-mark (SAIB:xSAIB:EtOH:NC 70:10:20:0.01) was radiolabelled with ^64^Cu as previously described, and at the time of injection the markers had an activity of 35 MBq/mL. All animal experiments were approved by the Danish National Animal Experiments Expectorate.

*Study setup:* NMRI female mice (Taconic, Lille Skensved, Denmark) were subcutaneously injected with 50 µL (1.75 MBq) marker on the right flank (n = 8) for NIR fluorescence imaging (NIR-FLI) of the fluorophore and PET-imaging of ^64^Cu over time. All eight mice were PET/CT and NIR-FLI scanned at 1 h, 4 h, 24 h and 48 h post injection, and three mice were NIR-FLI scanned after 2 weeks, 3 weeks and 4 weeks. Five mice were euthanized after PET/CT scanning and organs were collected and counted for 120 seconds on a well-counter (Wizard^2^, Perkin Elmer, USA).

*PET/CT-procedure:* Mice were anaesthetized using sevoflurane and placed on a heated bed for scanning procedures. PET scanning was done on a MicroPET (Focus 120, Siemens Medical Solutions, Malvern, PA, USA). The voxel size was 0.866 × 0.866 × 0.796 mm^3^, and in the centre field of view the resolution was 1.4 mm full width at half-maximum (fwhm). PET acquisitions times were 5 min for time points 1 h, 4 h and 10 min for 24 h, and 20 min for 48 h scan. Data were reconstructed with the maximum a posterior (MAP) reconstruction algorithm. For anatomical localization of activity, CT images were acquired with a small animal imaging CT imaging system (NanoScan microSPECT/CT, Mediso) by transferring the anesthetized mice on the same imaging bed between systems. After data reconstruction, PET and CT images were fused using the commercially available software (Inveon, Siemens Medical Solutions, Malvern, PA, USA). The emission scans were corrected for random counts and dead time. The PET and CT images were used to identify regions of tracer uptake in manually constructed and automatically segmented regions of interest (ROIs). ROIs were constructed around the gel, liver and kidney, and either %ID/gel or %ID/g was calculated.

*NIR-FLI procedure:* Mice were anaesthetized using isoflurane and NIR-FLI imaged using a dedicated small animal fluorescence imaging system and the corresponding software (IVIS^®^ Spectrum *in vivo* imaging system). Imaging was performed using a binning of 2, exposure time of maximum 120 seconds and excitation and emission wavelength of Ex: 745 nm and Em: 810-875 nm and reported as total fluorescence from a large manual ROI covering the area of the injected gel.

*Well-counting:* After the last PET scan time (48 h), five mice were euthanized, and organs collected for well-counting. The well counting protocol consisted of 120 seconds counting per organ sample, and the results were presented as average ± standard-error-of-mean (SEM).

*Marker volume:* The marker volume was obtained by automated segmentation procedure based on a CT contrast cut-off of 250 HU.

### Statistics

The statistics were calculated by one-way ANOVA with Tukey's multiple comparison post hoc test. Probability values below 0.05 were considered as statistically significant. All data are reported as statistical means ± SEM), unless otherwise stated.

## Results

### NC-mark absorbs and emits light in the near infrared region

**Figure 3:**
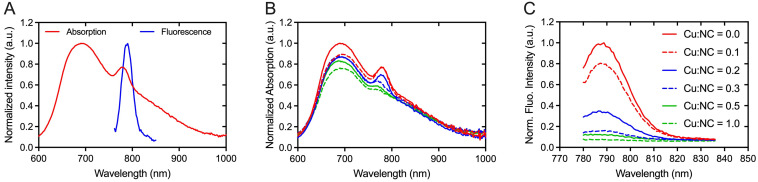
** Spectral properties of NC-mark.** (A) Absorption and emission spectra of NC-mark (SAIB:xSAIB:EtOH:NC 70:10:20:0.001, Ex. 700 nm). (B, C) Quenching of NC-mark upon spiking with Cu^2+^. (B) Normalized absorption and (C) normalized fluorescence emission spectra (Ex. 768 nm) of NC-mark (SAIB:xSAIB:EtOH:NC 70:10:20:0.001) spiked with different amounts of Cu^2+^. The Cu:NC ratios given in (B, C) present the molar ratio of Cu^2+^ and NC in each marker formulation.

Absorption and fluorescence spectra of NC-mark (SAIB:xSAIB:EtOH:NC 70:10:20:0.001 w/w) are presented in Figure 3. NC-mark displays a broad absorption band from 600-1000 nm with maxima at 698 nm and 786 nm and emits light from 750-850 nm with a maximum fluorescence emission intensity at 788 nm. The emission characteristics of NC-mark resembles the commonly used dye IRDye800CW (Ex/Em (774 nm/789 nm) and ICG (Ex/Em 807 nm/822 nm) 14 and have the potential to facilitate deep-tissue fluorescence imaging with high signal-to-background ratio 14. Additional phthalocyanine and naphthalocyanine dyes were tested as part of the marker composition yielding broader fluorescence emissions bands in the 700-900 nm range (SI, Figure S3).

### Radiolabeling of NC-mark with ^64^Cu^2+^ has negligible impact on the fluorescence intensity

In previous reports, Cu^2+^ was found to quench the fluorescence of organic dyes and fluorescent proteins 35, 36. Similar effects may occur for radiolabeling of NC-mark with ^64^Cu^2+^, and the Cu^2+^ mediated quench of the NC dye was therefore tested by adding variable amounts of CuCl_2_ to the marker. Both absorption (Figure 3B) and fluorescence emission intensity (Figure 3C) of the marker was found to decrease with an increased concentration of Cu^2+^, and the fluorescence emission was fully abolished at a 1:1 molar ratio of Cu and NC. These results indicate that complexes of Cu^2+^ and NC are efficiently formed upon Cu^2+^ spike into NC-mark.

For surgical procedures requesting preoperative PET scans and radio-guided localization, an activity of 1 MBq per 50 µL marker is sufficient, i.e. a concentration of 20 MBq ^64^Cu/mL at the time of injection is required. Radiotracers are usually labeled with an excess activity to account for decay before use. Radiolabeling of NC-mark with 100-300 MBq ^64^Cu/mL, corresponding to 5-15-fold excess, thus results in a copper concentration of 100-300 nM (specific activity > 1 TBq/µmol Cu) at preparation. At this radioactivity level, the NC dye is in 300-1000-fold excess to Cu, as 0.01 % (w/w) NC corresponds approximately to 100 µM NC. Under these conditions (Cu:NC ~ 0.001-0.003), the influence of ^64^Cu^2+^ labeling on the fluorescence intensity is considered negligible according to Figure 3C, as less than 1 % of the NC dye is occupied by Cu.

### The brightest NC-mark formulation with minimal self-quench

**Figure 4:**
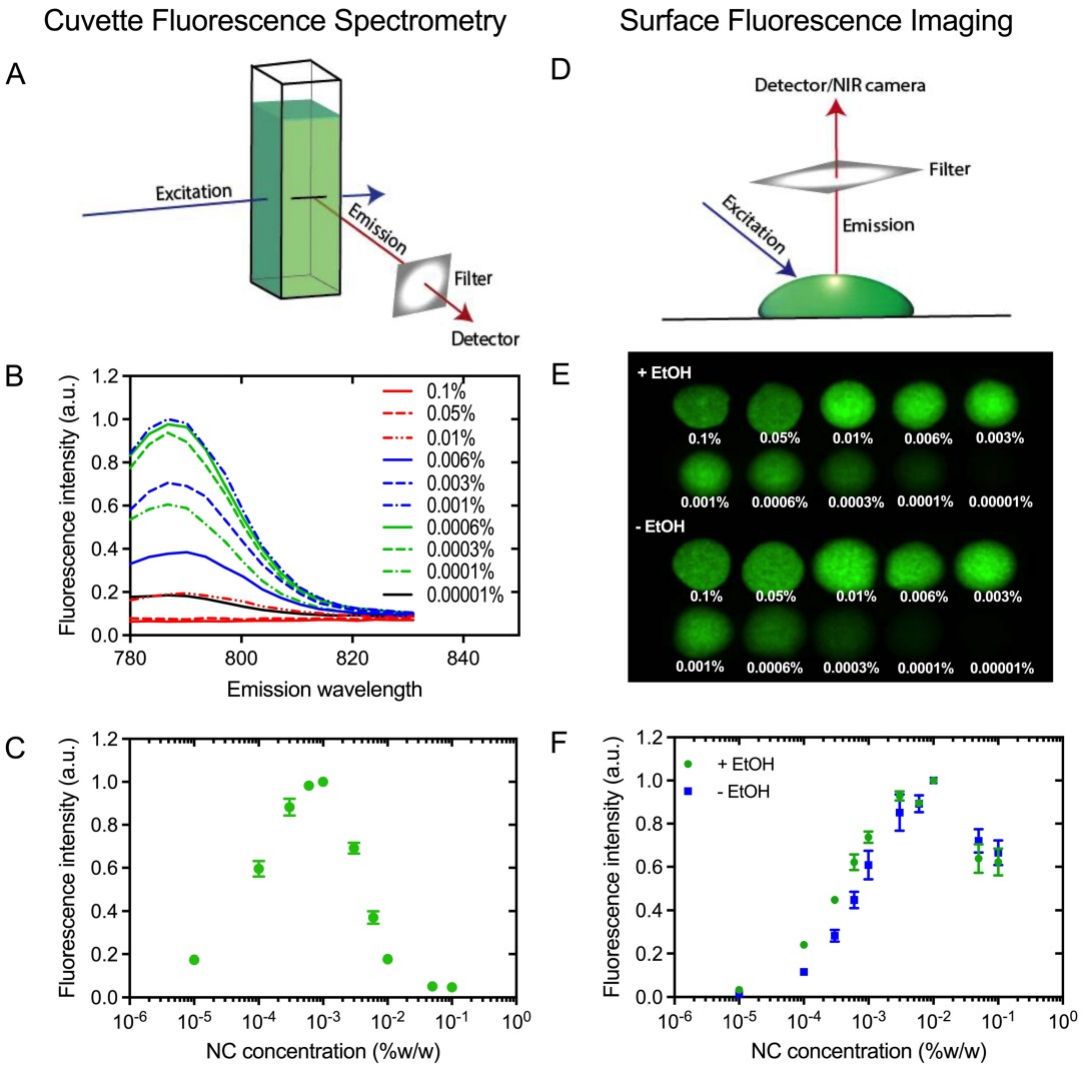
** Fluorescence self-quench analysis of NC-mark.** (A) Principle of cuvette fluorescence spectrometry. (B) Fluorescent emission spectra of NC-mark containing varying concentrations of the NC dye (Ex 768 nm). (C) Bulk fluorescence intensity at 788 nm as function of the NC dye concentration in the marker. (D) Principle of the surface fluorescent imaging assay. (E) Surface fluorescence images of markers with and without ethanol (Em/Ex 785 nm/800 nm). (F) Surface fluorescence intensity of markers with and without ethanol (shown in D). The results in C and F are presented as mean ± SEM (n = 3).

In order to obtain the brightest NC-marker visible at the greatest possible tissue depth, NC-mark was screened to identify compositions with minimal self-quench. A series of NC-mark compositions with different dye concentration were prepared and their fluorescence intensity were evaluated both using bulk fluorescence spectrometry and surface fluorescence imaging. In the cuvette-based fluorescence spectrometry assay (Figure 4A), emissions from bulk fluorophores were detected, whereas the surface fluorescence method primarily collected emissions from the surface of the marker (Figure 4D). Fluorescence emission spectra of NC-mark (SAIB:xSAIB:EtOH 70:10:20) are presented in Figure 4B, and corresponding changes in peak fluorescence intensity at 788 nm are presented in Figure 4C. The emission intensity of NC-mark was found to increase until 0.001 % (w/w) of the dye, after which it decreased. The shape and peak position of the emission spectra remained constant for all dye concentrations. In the surface fluorescence imaging assay (Figure 4D), images (Ex/Em 785 nm/800 nm) were collected (Figure 4E) with and without ethanol to assess the impact of the ethanol efflux that occurs as the marker sets in the tissue. Here the brightest formulation had a 0.01% (w/w) NC concentration independent of the ethanol content, indicating that *in situ* formation of the marker does not affect or lead to self-quenching. The concentration at which self-quenching occurs was found to be 10-fold higher for the surface-, compared to the bulk fluorescence, assay. This finding may be reasoned by the shorter distance that the photons travel in the marker when emitted from the marker surface, leading to lower absorption and scattering induced attenuation, and hence more effective emissions at higher NC concentrations. As the surface fluorescence assay best resemble the clinical imaging setup, a concentration of 0.01 % (w/w) NC was chosen for further evaluation *in vivo*.

### Efficient ^64^Cu radiolabeling of NC-mark

Radiolabeling of NC-mark was achieved by a simple mixing step, in which the liquid marker formulation was added to dry [^64^Cu]CuCl_2_. Hereafter, ^64^Cu was complexed by displacing two central hydrogens in the tetrapyrrolic macrocycle of NC 25. In order to confirm the formation of the ^64^Cu-NC complex, a non-radioactive reference compound (Cu-NC) was prepared by an analogous method to the radiolabeling process. The mass of the formed Cu-NC complex was confirmed by MALDI-TOF and corresponded with the target structure (SI, Figure S1), and a retention factor of Rf = 0.8 was determined by TLC. Effective radiolabeling of NC-mark was obtained within 2 hours, with a high radiochemical purity (RCP) of >95%. Radio-TLC analysis supported the formation of the ^64^Cu-NC complex yielding a retention factor (R*f*) that was equivalent to that of the reference compound.

### Stable and efficient radiolabeling of NC-mark

**Figure 5:**
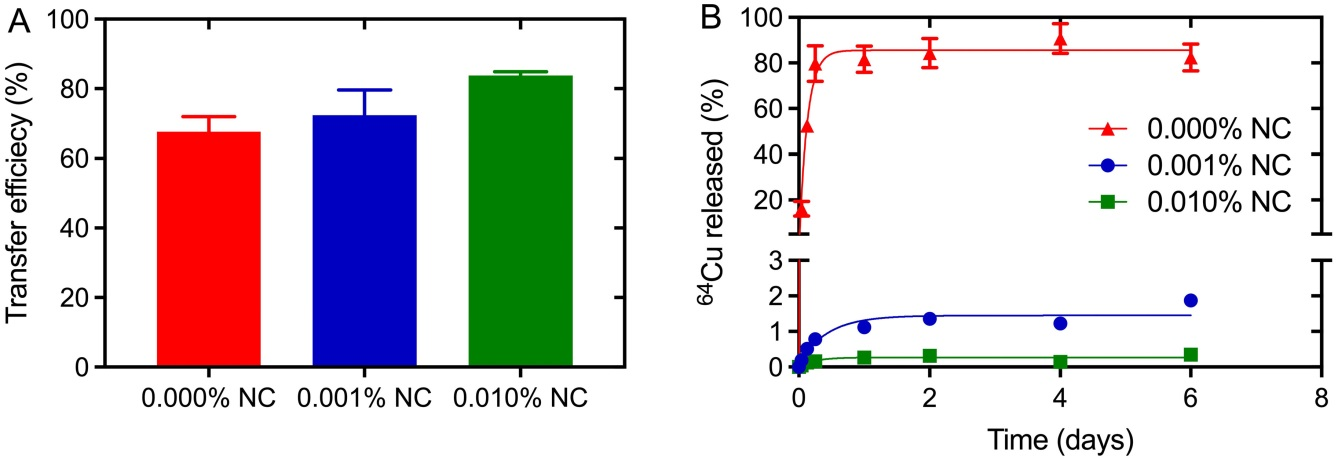
**Transfer and retention of activity upon radiolabeling of NC-mark.** Characterization of ^64^Cu transfer efficiency (A) and *in vitro* marker retention (B) in NC-mark (SAIB:xSAIB:EtOH 70:10:20) with varying NC concentration. The transfer efficiency was determined using a dose-calibrator, and the ^64^Cu released from the marker was quantified by LSC. The initial activity concentration of the markers was 300 MBq/mL, and the results are reported as mean ± SEM (n = 3).

Efficient radiolabeling procedures and marker stability in tissue is a prerequisite for the use of NC-mark in surgical procedures. The ^64^Cu transfer efficiency and ^64^Cu retention was therefore analyzed for markers with increasing NC concentration (0, 0.001 and 0.01 % w/w) to assess NCs propensity for chelating and trapping ^64^Cu. The transfer efficiency, presented in Figure 5A, was found to be ~70% even for the marker matrix (SAIB:xSAIB:EtOH) without NC. This indicates coordination of Cu^2+^ to marker constituents, potentially to oxygens present on the ester groups of SAIB. The transfer efficiency increased further from 68 % to 72 % to 84 % for marker formulations containing 0.001 % (w/w) and 0.01 % (w/w) NC, respectively, substantiating that NC complex formation increases the solubility of ^64^Cu in the marker formulation. Quantitative transfer of ^64^Cu could possibly have been obtained by further increase in NC concentration. This would however have led to quenching of the NC fluorescence, and was therefore not pursued.

Premature leaching of ^64^Cu or NC dye from the marker would potentially lead to loss of positional accuracy during the surgical procedure. NCs are highly hydrophobic 27 and as expected no NC was detected in the release media in the *in vitro* release assay (SI, Figure S2). Leaching of ^64^Cu from NC-markers containing either 0, 0.001 or 0.01 % (w/w) NC was tested by injection of these into release media containing 10 mM ISO-TRIS, 1.0 mM EDTA, and 5.0 mM stealth liposomes. EDTA was added as scavenger of ^64^Cu to test the stability of the Cu-NC complex, and the liposomes acted as hydrophobic reservoir for NC. Nearly complete release of ^64^Cu was obtained for markers without NC (Figure 5B), whereas markers containing 0.001 % (w/w) or 0.01 % (w/w) NC displayed high retention of ^64^Cu. A 10-fold increase in NC concentration (0.001 % w/w to 0.01 % w/w) reduced the ^64^Cu release from <2 % to <0.4 %. These results clearly show that the marker matrix acts as an efficient but labile chelator for Cu, whereas ^64^Cu in the presence of NC forms ^64^Cu-NC chelates with high stability, capable of defying an EDTA challenge of 1 mM over several days.

### Excellent *in vivo* tumor retention and photostability of ^64^Cu radiolabeled NC-mark

**Figure 6:**
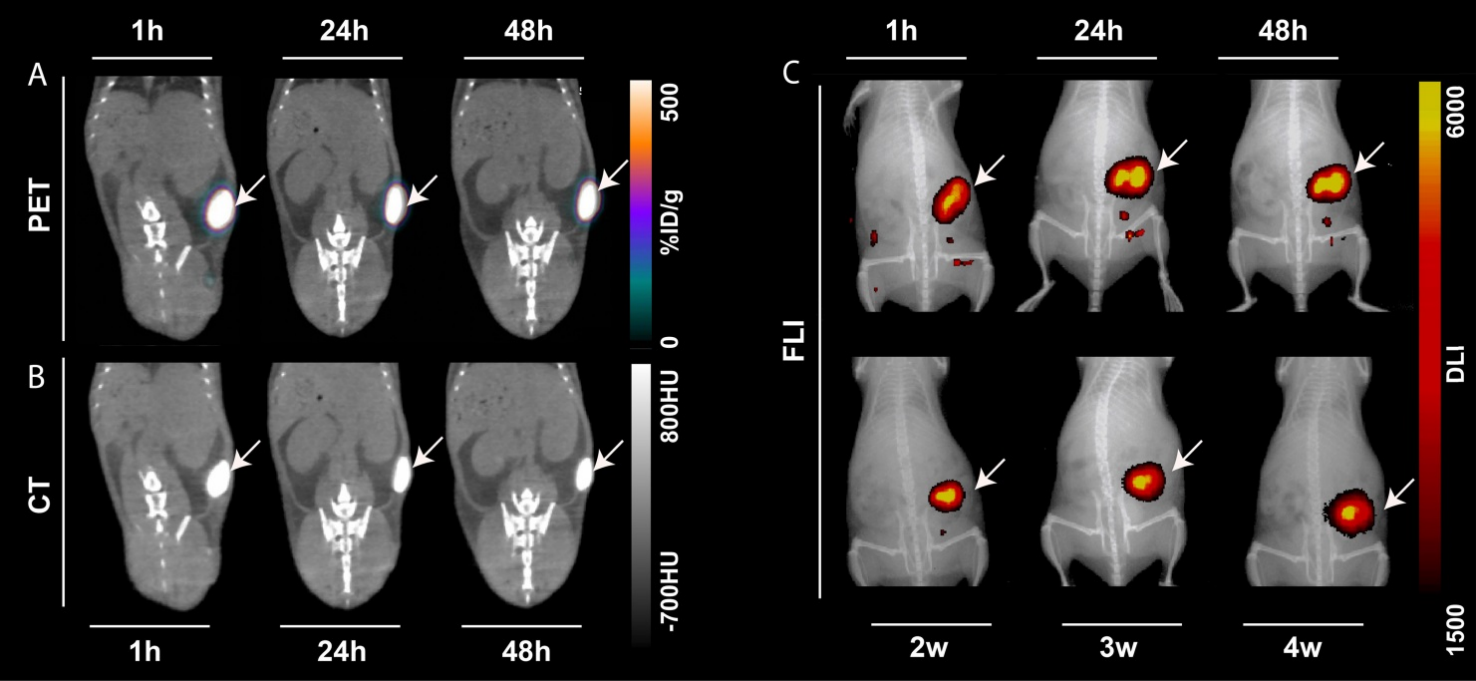
** PET/CT, CT and NIR-FLI of the ^64^Cu-radiolabeled naphthalocyanine based liquid marker.** Representative PET, CT and FLI images of one mouse injected with 50 µL ^64^Cu-NC-mark as a function of time. (A) Coronal PET/CT images, (B) CT images. From left to right: 1 hour, 24 hours and 48 hours post injection. (C) Fluorescence (FLI)/x-ray overlay images (Ex/Em 745 nm/ 780-800 nm). Top row from left to right: 1 hour, 24 hours and 48 hours, low row from left to right: 2 weeks, 3 weeks and 4 weeks post injection. The white arrows indicate the injection position of the marker.

**Figure 7:**
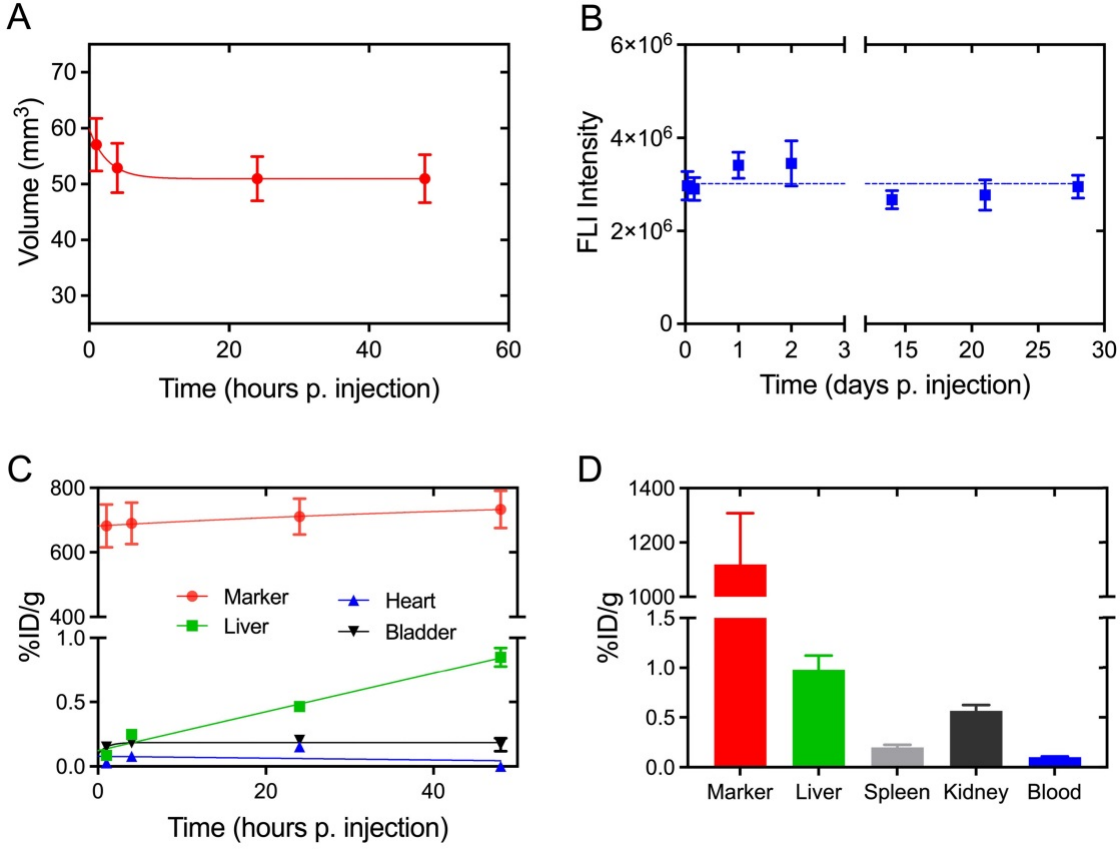
** Quantitative assessment of the *in vivo* performance of ^64^Cu-NC-mark based on PET/CT and NIR-FLI images.** (A) Marker volume based on CT-contrast segmentation, (B) marker fluorescence intensity determined via NIR-FLI images. (C) PET based accumulation and retention of ^64^Cu in the marker, liver, heart and bladder given as % injected dose per gram (%ID/g) as function of time. (D) Well counting data for the marker, liver, spleen, kidney and blood at 48 h post injection. Results are presented as mean ± SEM, (A) n = 8, (B) n = 8 (0 - 48h), n = 3 (1 - 3 weeks), (C) n = 5 and (D) n = 5.

The performance of ^64^Cu-radiolabeled NC-mark (^64^Cu-NC-mark) was evaluated *in vivo* by subcutaneous injection into the right flank of NMRI mice (n = 8). PET, CT and NIR-FLI images of the marker at 1 h, 24 h and 48 h post injection (Figure 6) show high retention of ^64^Cu, x-ray contrast and fluorescence within the marker, i.e. essentially all signals co-locate with the position of the marker. NIR-FLI images were further recorded 2-, 3- and 4-weeks post injection, and no visible change in marker brightness or leaching of the NC dye (Figure 6C) was found. The *in vivo* stability of ^64^Cu-NC-mark was further quantified by ROI/VOI-analysis of the CT, PET and fluorescence images. Here, NC-mark (Figure 7A) displayed a volume reduction of ~10 % within the first 4 hours, after which the volume was stable. This loss in volume is attributed to ethanol efflux, and is consistent with previous studies 18, 30. The NIR-FLI intensity (Figure 7B) remained high and stable (90-116 % of the initial intensity) throughout the study period underlining the high marker retention, excellent quantum yield and photostability of the NC dye. The excitation and emitted fluorescence light from the markers were however attenuated by the skin and tissue, and the observed variations in NIR-FLI intensity may reflect varying skin and tissue thickness caused by minor changes in position of the animals during the imaging procedures and/or stretching of the skin. Changes in shape of the individual markers may also have contributed to these intensity fluctuations.

Analyses of the PET images demonstrate high retention of activity within ^64^Cu-NC-mark (Figure 7C) from 1 hour to 48 hours post injection. The marker activity concentration shows an increasing trend (although not significant) from 680 ± 70 % ID/g (1 h) to 730 ± 60 % ID/g (48 h), reflecting the loss of marker volume and hence confinement of the activity in a smaller volume. The high retention of activity *in vivo* (Figure 7C) is fully supported by the limited loss of ^64^Cu found in the leaching assay (Figure 5B). Over time, minute quantities of activity were lost from the marker, and were mainly located in the liver (0.85 ± 0.07 %ID/g, 48 h) and bladder (0.17 ± 0.05 %ID/g, 48 h). Free [^64^Cu]CuCl_2_ is known to accumulate in the liver of mice 37, 38 whereas NC is highly hydrophobic and biodistribution has primarily been reported for water soluble sulphonated analogues 22-24. The activity observed in the liver is therefore most likely caused by release of ^64^Cu from the marker, and not by accumulation of ^64^Cu-NC. After 48 hours, 5 mice were sacrificed, and organs of interest were well-counted (Figure 7D). Both PET and well-counting data displayed high retention of activity within the marker and minimal loss of activity to the liver, spleen, and kidney. Lower marker radioactivity concentrations were however obtained from the PET analysis compared to well-counting data. This difference may be explained by a partial volume effect when determining the gel volume and gel VOI based on CT contrast-based segmentation.

## Discussion

The presented NC-mark formulation has optimal features as soft tissue marker for intraoperative NIR- and radio-based surgical guidance and is injectable by small-gauge needles. These imaging properties combined with the gel-forming liquid technology make NC-mark an attractive alternative to current clinical markers. The marker is intrinsically visible in ultrasonography (US) and magnetic resonance imaging (MRI), and has x-ray contrast due to the incorporation of the xSAIB construct 32, 39. xSAIB is a mixed isobutyrate, triiodophenoxyacetate sucrose ester derivative, which contains six aromatically bound iodine per molecule providing the high electron density required for CT imaging and 2D x-ray fluoroscopy 40. These image features allow for preoperative localization via US/CT or 2D x-ray fluorescence guidance during injection of the marker. NC-mark further contains a multifunctional naphthalocyanine dye that efficiently chelates the PET radionuclide ^64^Cu and fluoresces in the NIR-I range at 770-810 nm. The currently used NC dye is part of a larger family of phthalo- and naphthalo-cyanines that all have a similar tetrapyrrolic macrocyclic core-structure enabling radionuclide chelation. The emission and excitation range of NC-mark can therefore easily be tuned by exchange of the dye, and thereby be adapted to fit the spectral specification of most FDA approved and CE marked NIR surgical cameras 41. Contrary to the currently approved MB and ICG NIR dyes that are dispersed upon injection 9, 10. NC-mark retains the fluorescent dye in high concentration at the site of injection with no detectable leaching. In combination with the high photostability of the phthalo- and naphthalo-cyanine dyes 42 this feature, ensures a high signal-to-noise ratio and optimal guidance of the surgeon for weeks after placement of the marker.

NC-mark can be radiolabeled with ^64^Cu in high radiochemical purity and with high transfer efficiency by a simple mixing step that can be conducted in hospital radiopharmacy facilities. This simplifies the distribution of ^64^Cu-NC-mark as it can be produced on site from a vial of NC-mark and a batch of dry [^64^Cu]CuCl_2_. Detection of annihilation photons from ^64^Cu positron emissions using handheld high energy gamma detectors or intraoperative laparoscopic gamma tracing 16, 28 allows the surgeon to locate lesions at greater tissue depths than what can be detected by a NIR camera. Ultimately, the surgeon can initiate the surgical procedure using radio-guidance to provide accurate tracking and dissection during the surgical approach and switch to NIR image guidance for high resolution tracking once the target is visible by NIR. The latter approach may be highly relevant in robotic surgery where NC-mark could serve as beacons or waypoints even for deeply situated lesions. Marker systems similar to NC-mark has previously been described where NIR fluorescence and radiolabeling was achieved through incorporation of a custom synthesized Cy7.5 fluorophore and ^64^Cu radiolabeling via ionophores 33. In that study, the use of ionophores yielded a less stable and less convenient radiolabeling procedure compared to radiolabeling of NC-mark. ^64^Cu has a short half-life of 12.7 hours and may be replaced by other radionuclides if guidance at longer or shorter timescales is requested. Examples of alternative radionuclides are ^99m^Tc (T_½_ = 6.0 h), ^111^In (T_½_ = 2.8 d), ^67^Ga (T_½_ = 78.3 h), and ^68^Ga (T_½_ = 271 d) 26. Still, NC-mark labeled with ^64^Cu has a sufficient activity for surgical guidance up to 2-4 half-lives, i.e., 1-2 days after injection of the marker. The efficient retention of both the NIR dye and ^64^Cu within NC-mark enables precise co-localization of the NIR/PET signals, as well as the intrinsic US and MRI contrast, which may be utilized for co-registration purposes. The high integrity of NC-mark confers high safety and reduced side effects, as it limits harmful exposure to the NIR dye or ^64^Cu for the patient. In addition, the coalescent properties of the marker allow for full recovery of material during the surgical procedure, preventing prolonged exposure to the radioactive source. This is in contrast to administration of the MB or ICG NIR dyes or ^99m^Tc nanoparticles where the patient is exposed to a dispersing tracer. Radio-guided localization of tissue markers may be achieved even at low kBq to MBq activity levels, far below those administered for standard radiotracers. This minimizes the risk of unwanted radiation doses to either the patient or surgeon.

Contrary to most soft tissue markers, such as hook-wires, low activity brachytherapy or gold seeds, NC-mark is a liquid that may be injected through standard thin needles and is compatible with modern endoscope technology. This enables accurate and precise positioning of NC-mark in nearly any tissue or site in the body, and further reduces patient discomfort.

## Conclusion

We have successfully developed a soft tissue marker for intraoperative NIR and radio-guided localization of lesions. The marker is designed to guide the surgeon in real-time for resection of the smallest lesions that can be identified on diagnostic imaging. Contrary to tumor targeted NIR/PET-probes, NC-mark does not require expression of tumor specific receptors, it is visible on multiple image modalities such as US/MRI/CT and PET, it is highly versatile, and allows for marking of noncancerous lesions.

NC-mark is a liquid based on SAIB and ethanol and can be administered to almost any site in the body with high positional precision and accuracy, using state of the art image-guided injection technologies. Upon administration, the liquid solidifies and forms a gel-like depot at the site of injection, where the NIR signal and ^64^Cu activity are demonstrated to be effectively confined, through the use of the dual functional naphthalocyanine dye. High precision injection and the effective retention of marker constituents are key elements for accurate localization of the target lesion and are unique features of NC-mark. The position of NC-mark relative to the target lesion can be verified preoperatively to enhance the success rate for positive resection using a range of clinical image modalities (CT/PET/US/MRI). For surgical procedures, the surgeon may initiate localization of the marker (and lesion) using radio-guidance and proceed to NIR image guidance once the target is in sight.

NC-mark is composed of biocompatible materials, and similar compositions have been successful in clinical tests 19. Radiolabeling of NC-mark can be achieved by a simple mixing procedure, and can be conducted at local radiopharmacies or hospitals, which is a major advantage for obtaining regulatory approval. The current number of ^64^Cu suppliers worldwide may initially limit the future use of NC-mark but use of more abundant radionuclides or adaptation of ^64^Cu production at local hospital cyclotron facilities may alleviate this. NC-mark may further have applications within brachytherapy by loading of therapeutic radionuclides such as ^67^Cu, or in photodynamic therapy, or in emission guided radiation therapy.

## Supplementary Material

Supplementary figures and tables.
