# Supplementary material for: Carbohydrate based biomarkers enable hybrid near infrared fluorescence and 64Cu based radio-guidance for improved surgical precision
Source: Nanotheranostics. 2021 May 17;5(4):448–60. doi: 10.7150/ntno.60295 (PMC8156217; doi:10.7150/ntno.60295)
Supplement: Supplementary file 1 — Supplementary figures and tables. [file ntnov05p0448s1.pdf]

Supporting Information for

Carbohydrate based biomarkers enable hybrid near infrared fluorescence and ^{64}Cu based radio-guidance for improved surgical precision

Wenbo Wang[1,5,#], Anders Elias Hansen[1.5.#], Hongmei Sun[3,#], Frederikke Petrine Fliedner[2], Andreas Kjaer[2], Andreas I. Jensen[4,5], Thomas L. Andresen[1,5], Jonas R. Henriksen[1,5,*]

[1]*Technical University of Denmark, Department of Health Technology, Building 423, 2800 Lyngby, Denmark*

[2]*Rigshospitalet and University of Copenhagen, Dept. of Clinical Physiology, Nuclear Medicine & PET, Cluster for Molecular Imaging, 2100 Copenhagen, Denmark*

[3]*School of Bioengineering and Food, Key Laboratory of Fermentation Engineering, (Ministry of Education), Key Laborat ory of Industrial Microbiology in Hubei, National '111' Center for Cellular Regulation and Molecular Pharmaceutic,Hube i province Cooperative Innovation Center for Industrial Fermentation, Hubei University of Technology, Wuhan 430068, China*

[4]*Technical University of Denmark, The Hevesy Laboratory, Department of Health Technology,* 4000 Roskilde, Denmark

[5]Center for Nanomedicine and Theranostics, Technical University of Denmark, 2800 Lyngby, Denmark.

#Contributed equally to the work

*Corresponding Author, email:jhen@dtu.dk

S1: Formation of a non-radioactive reference Cu-NC complex (Cu - 2,11,20,29-tetra-tert-butyl-2,3-naphthalocyanine)

A non-radioactive reference of the Cu-NC complex was prepared, analyzed, and compared to the radioactive counterpart (^{64}Cu-NC) by TLC.

Method and results:

A chloroform solution with NC (1mL, 1mg/mL) was added to 0.02 mg $CuCl_2 \cdot 2H_2O$ (molar ratio Cu^{2+}:NC 10:1). The resulting mixture was magnetically stirred at 55 °C for 2 hours. The Cu-NC product and NC dye alone were analyzed by MALDI-TOF MS (Bruker Reflex, Bruker Daltonics, Billerica, MA, USA): Calc. M: 939.2 Da., Obs. M: 939.2 Da. (NC dye). Calc. M: 1000.7 Da., Obs. M: 1000.0 Da (Cu-NC complex) (Figure SI-1). 1 µL of the Cu-NC reference in chloroform was spotted on silica gel 60 F254 plates (Merck) and a solution of $CHCl_3$:MeOH:AcOH 98:1:1 was used as eluent. The R*f* of resulting Cu-NC complex was about 0.8.

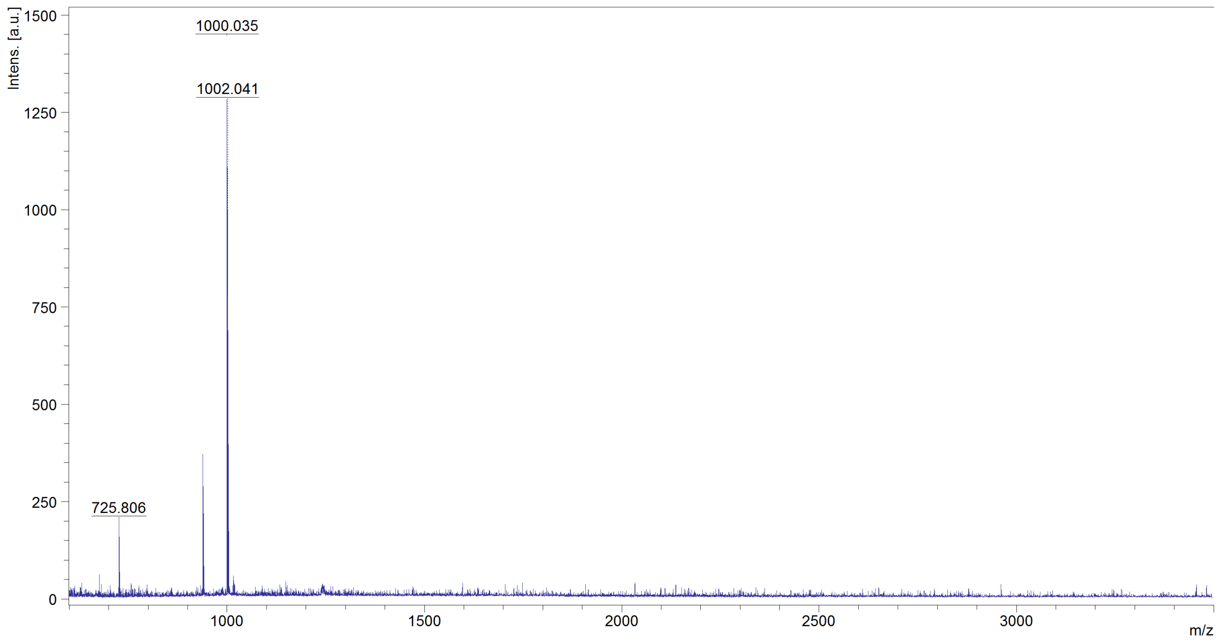

Figure S1: Mass spectra of the formed Cu-NC reference complex. The peak of 1000 presents the mass of Cu-NC complex. The peak of 1002 presents the mass of Cu-NC complex + 2H.

S2: In vitro release of NC from a gel marker

Method and results:

The release of NC dye from the NC-mark was investigated *in vitro*, by injecting 300 µL of NC-mark (0.1% w/w NC) into 5 mL of phosphate buffer saline (PBS, 5 mM, 150 mM NaCl, pH 7.0). The sample was following stored in the dark at 37°C, and dye release was monitored by UV-vis spectroscopy after 1, 3, 6 hour and 1, 2, 4, 6 days. UV-vis spectra of 0.5 ml PBS release buffer sample was recorded in quartz cuvettes from 200 nm to 850 nm using a Nanodrop 2000c (Thermoscientific, US) spectrophotometer. A PBS solution (release standard) corresponding to 10% release of NC was prepared from a NC solution in acetonitrile (0.05 mg/mL). At preparation, NC partially precipitated after dilution into PBS. The supernatant of the 10% release standard solution was taken after storage at room temperature overnight. The concentration of the supernatant thus contains less NC than intended, and therefore represents a lower estimate of the 10% release standard.

The UVvis absorption of the release samples (6D-1, 6D-2, 6D-3) was found to be negligible and nearly non-detectable compared to the 10% release standard solution (Fig. S2), which indicate minimal release of NC over the period of 6 days.

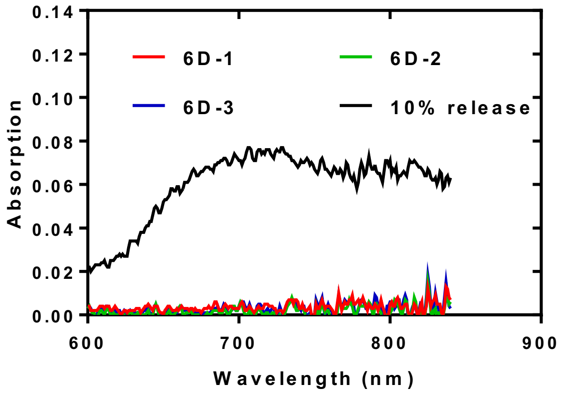

Figure S2: *In vitro* release of NC dye from the marker. UVvis spectra of NC in the PBS release media on day 6 after injection into buffer (conducted in triplicate). A standard corresponding to 10% release was included for reference.

S3: Characterization of alternative dual functional naphthalocyanine or phathalocyanine dyes for extended absorption and emission range of NC-mark

Methods

Two dyes, 5,9,14,18,23,27,32,36-octabutoxy-2,3-naphthalocyanine (NC-2) and 2,9,16,23-tetra-tert-butyl-29*H*,31*H*-phthalocyanine (PC-3) were purchased from Sigma Aldrich. The structures are presented in Scheme S1.

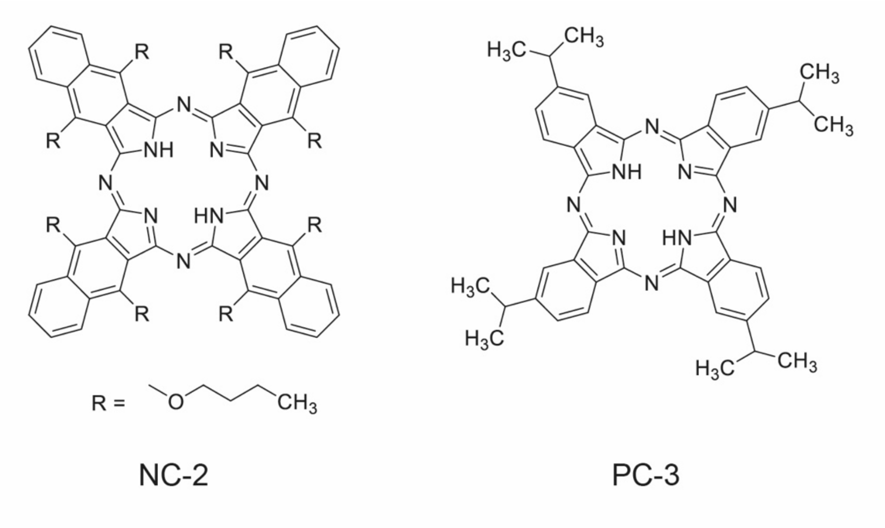

Scheme S1. Chemical structures of NC-2 (5,9,14,18,23,27,32,36-octabutoxy-2,3-naphthalocyanine) and PC-3 (2,9,16,23-tetra-tert-butyl-29*H*,31*H*-phthalocyanine).

Preparation of NC-2 and PC-3 markers

NC-2 marker formulation: SAIB was heated to 70°C, and SAIB was poured into a glass vial. SAIB (8.0 g) and benzyl alcohol (BA, 2.0 g) was mixed and sonicated for 30 minutes to obtain a transparent and homogeneous SAIB:BA formulation (SAIB:BA 80:20).

A solution of NC-2 dissolved in chloroform (100 µL, 1 mg/mL) was pipetted into a glass vial, and the chloroform was evaporated at room temperature under nitrogen flow. Subsequently, marker formulation (SAIB:BA 80:20, 1.0 g) was added into the vial to achieve a NC-2 concentration of 0.01% for absorbance measurement. The resulting mixture was sonicated at 70°C for 15 minutes and following by vortexing. The NC-2 marker formulation was further diluted using SAIB:BA 80:20 to a NC-2 concentration of 0.005% for fluorescence emission measurement.

PC-3 marker formulation: SAIB was heated to 70°C, and SAIB was poured into a glass vial. SAIB (7.0 g), xSAIB (1.0 g) and ethanol (2.0 g) was mixed and sonicated for 30 minutes to obtain a transparent and homogeneous SAIB:xSAIB:ethanol formulation (SAIB:xSAIB:ethanol 70:10:20).

A solution of PC-3 dissolved in chloroform (50 µL, 1 mg/mL) was pipetted into a glass vial, and the chloroform was evaporated at room temperature under nitrogen flow. Subsequently, marker formulation (SAIB:xSAIB:ethanol 70:10:20, 1.0 g) was added into the vial to achieve a PC-3 concentration of 0.005% w/w for fluorescence emission measurement. The resulting mixture was sonicated at 70°C for 15 minutes and followed by vortexing. The PC-3 marker formulation was further diluted using SAIB:xSAIB:ethanol 70:10:20 to a PC-3 concentration of 0.001% for absorbance measurement.

UV-vis absorbance measurements

Each marker solution (0.2 mL) was pipetted into a 96-well plate, and the UV-vis spectrum (400 – 1000 nm) was recorded by a multimode microplate reader (Spark®, Tecan) with bandwidth of 3.5 nm.

Fluorescence emission measurements

Each marker formulation (1.0 mL) was transferred to a quartz cuvette (Helma, 10mm light path), and the fluorescence spectrum was collected by a fluorescence spectrometer (OLIS DM 45) with excitation/emission bandwidth of 26 nm and integration time of 0.2 seconds. An excitation wavelength of 650 nm was used for the PC-3 marker formulation. An excitation wavelength of 750 nm was utilized for NC-2 marker formulation.

Results

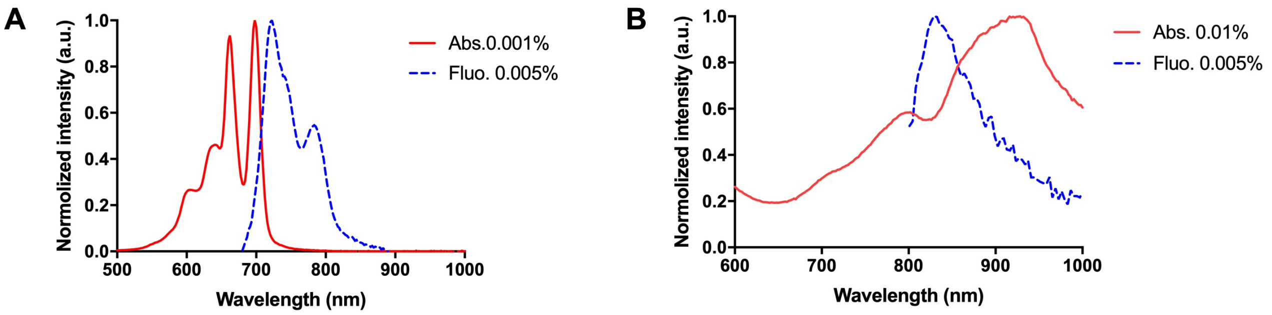

Figure S3. Absorbance and fluorescence spectra of NC-2 and PC-3 dissolved in marker formulations. (A) Fluorescence and absorbance spectra of PC-3 dissolved in in SAIB:xSAIB:EtOH 70:10:20 marker formulation. (B) Fluorescence and absorbance spectra of NC-2 dissolved in SAIB:BA 80:20 marker formulation.
